# Self-esteem, job insecurity, and psychological distress among Chinese nurses

**DOI:** 10.1186/s12912-021-00665-5

**Published:** 2021-08-10

**Authors:** Yun Liu, Chunyan Yang, Guiyuan Zou

**Affiliations:** 1 Liaocheng People’s Hospital, Liaocheng, China; 2 Shandong Mental Health Center, 49Wenhua East Road, 250012 Jinan, P.R. China

**Keywords:** Job insecurity, Self-esteem, Psychological distress, Mediation, Nurse, China

## Abstract

**Background:**

Many studies investigate the variables relating to psychological distress among nurses, but little is known about the underlying mechanism(s) among job insecurity, self-esteem, and psychological distress.

**Aims:**

This cross-sectional study examines the prevalence of psychological distress among nurses and the relationships among job insecurity, self-esteem, and psychological distress; it also explores how self-esteem might mediate between job insecurity and psychological distress.

**Methods:**

Questionnaires that assess job insecurity, self-esteem, and psychological distress were collected from 462 nurses in a tertiary hospital in Shandong Province, China.

**Results:**

Our results show an 83.3 % prevalence rate for psychological distress among nurses. Regression analysis results show that job insecurity positively correlates with psychological distress, explaining 17.5 % of the variance in psychological distress. Mediation analysis results show that self-esteem partially mediates the effect of the two dimensions of job insecurity on psychological distress.

**Conclusions:**

Psychological distress is prevalent among Chinese nurses. Nursing administrators should take effective measures to improve self-esteem and reduce the negative impacts of job insecurity on nurses, including psychological distress.

## Introduction

Over the last decade, psychological distress among nurses has been a research focus among researchers in occupational health [1–3]. Several researchers have found not only that psychological distress is a risk predictor of psychosomatic function and quality of life [1, 4]: it also gives rise to poor job performance and high turnover rates, both of which undoubtedly affect nursing professionalism and quality of care [5, 6]. Today, ongoing changes in the healthcare profession—such as fiercer competition among hospitals, changing employment relations, increased workloads, and deficiencies in both technical knowledge and decision-making autonomy—have resulted in organizational and personal challenges [7, 8]. These challenges—coupled with rapid economic development, general population aging, the growing burden of chronic diseases, unreasonable expectations among administrators and patients, and unsafe working conditions or practice environments—have led to increased psychological distress and caused nurses to feel insecure about their jobs [9, 10].

“Job insecurity” is defined as the subjective perception of a potential threat to the continuity of one’s current job [11]. “Quantitative job insecurity” refers to an overall concern about the continued existence of the job in the future [12, 13]. “Qualitative job insecurity,” on the other hand, refers to perceived threats of impaired quality in the employment relationship, such as a reduction in income, a lack of career opportunities, and the deterioration of working conditions [12]. Job insecurity is a form of job stress that undoubtedly affects professional performance and psychosomatic health [14–16]. In addition, researchers have found a significant relationship between job insecurity and sleep disorders, psychological distress, burnout, job satisfaction, and problems with family relationships [16, 17]. While a large number of population-based empirical studies have investigated the detrimental effects of job insecurity on work-related attitudes and health and well-being outcomes, studies on the relationship between job insecurity and psychological distress among nurses are limited [18, 19].

Self-esteem has also attracted research attention as a psychosocial determinant of nurse health [20, 21]. Self-esteem is defined as an individual’s feelings regarding their personal worth, competence, and suitability [22, 23]. Studies have shown that self-esteem is one of the strongest predictors of life satisfaction, and it negatively correlates with psychological distress (especially in the forms of anxiety, depression, interpersonal problems, and high values in terms of the general severity index of symptom disorders) [24–26]. Thus, self-esteem is important to an individual’s psychological development. Self-esteem is a psychological determinant that can help individuals effectively cope with stressful dilemmas [23, 27]. Previous surveys found that self-esteem negatively correlates with perceived stress [22], and that it is worthwhile to consider self-esteem as an intermediary variable [21, 28, 29]. For example, Wang revealed that an individual’s self-esteem could mediate the relationship between occupational stress and well-being [27]. Based on these facts, we assert that self-esteem might mediate the effects of job insecurity among nurses, especially psychological distress.

## Objectives

This cross-sectional study looks to achieve the following aims. First, it investigates the prevalence of psychological distress among Chinese nurses; second, it examines the relationships among job insecurity, self-esteem, and psychological distress; finally, it explores the mediation of self-esteem on job insecurity and psychological distress.

## Methods

### Study design, setting, and sampling, and ethical considerations

We undertook our cross-sectional study in a tertiary hospital in Shandong Province, China, from July 2018 to September 2018; we used convenience sampling to survey registered nurses. We calculated the sample size as per the following formula: N = (*µα* S/δ)^2^ (where *µ*_*α*_ = 1.96, and the S/δ value = 10 according to the pilot study); in so doing, considering 20 % nonresponse rates, we found the minimum sample size was 461. A total of 480 nurses employed at the hospital were invited to participate in the survey, to which 462 of them responded (response rate: 96.25 %). The inclusion criteria for the participants were as follows: (a) held a nursing practice license in the People’s Republic of China, and (b) were directly involved in a nursing unit (i.e., internal medicine department, surgical department, intensive care unit, emergency department, or gynecology department).

Our study design and procedures were approved by the ethics committee of Shandong Mental Health Center (2018R23). These procedures were conducted in accordance with the ethical standards of the 1964 Helsinki Declaration. All participants were informed of the purposes and procedures of the study and provided written informed consent.

### Data collection procedures

Prior to formal execution, we telephoned each head nurse and explained the purpose of our study, and invited them to promote our research among the nurses. Data collection was conducted separately in each participating hospital ward.

Participants were informed orally and in writing of the study’s purpose, along with information on their rights as participants and the anonymity of this research project; we invited them to participate in our survey (i.e., a self-administered hard-copy questionnaire), which they were instructed to complete in a staff room or health education room following their daily work. The questionnaire required approximately 10–15 min of completion time. Ultimately, 462 nurses returned the completed questionnaires (response rate: 96.25 %). The most frequently cited reason for refusal was a lack of interest in the survey.

### Participants’ sociodemographic characteristics

The sociodemographic characteristics of the participants included age, gender, educational level, marital status, economic status, years of work experience, and job type.

### Rosenberg self-esteem scale

The Rosenberg self-esteem scale (RSES), a measure of individual self-esteem, comprises 10 items such as “All in all, I am inclined to feel that I am a failure.” [30]. The respondents rated each item according to the extent to which they agreed with the statement therein, on a four-point Likert scale whose values ranged from 1 (*do not agree at all*) to 4 (*agree completely*). Answers were summed to obtain a total score ranging from 10 to 40, with a higher score indicating a higher level of self-esteem. Factor analysis has shown the Chinese version of the RSES to be a valid single structure, and its alpha reliability for Chinese nurse sample ranges from 0.83 to 0.85 [31, 32]. In this survey, the Cronbach’s α of the RSES is 0.79.

### Kessler-10 rating scale

We used the Kessler-10 (K10) rating scale developed by Kessler and Mroczek to evaluate the level of mental health among the nurses [33]. This scale consists of 10 items; we obtained a response to each item via a five-point Likert scale with values ranging from 1 (*not at all*) to 5 (*extremely*). The total score for each participant ranged from 10 to 50, with a higher total score indicating a higher level of psychological distress. A total score ≥ 16 indicates psychological distress. Many studies support the psychometric properties of the K10 as a measure of mental health among clinical and nonclinical samples [34, 35]. Moreover, the K10 has also being found to be reliable and generate valid results among Chinese nurses [36], and in the current study its Cronbach’s α is 0.92.

### Job insecurity scale

We evaluated job insecurity using a seven-item scale developed by Hellgren [12]. The scale consists of two dimensions—one containing three items, and the other four—and it was designed to evaluate quantitative and qualitative insecurity. Participants were required to rate each item on a five-point Likert scale ranging from 1 (*strongly disagree*) to 5 (*strongly agree*). A combination of higher quantitative and qualitative insecurity indicated greater job insecurity. The scale has been proven valid (χ2/df = 2.283, CFI = 0.979, NFI = 0.974, RMSEA = 0.076) and reliable in measuring job insecurity experienced by clinical nurses [37]. (In the current study, the Cronbach’s α is 0.730, 0.949, and 0.903 for the overall job insecurity scale and the subscales.) In the current study, the Cronbach’s α for the quantitative and qualitative insecurity is 0.76 and 0.87, respectively.

### Data analysis

We used the Statistical Package for the Social Sciences (SPSS) software (v22) (IBM Corp., Armonk, NY, USA) in all analyses. We calculated descriptive statistics for psychological distress, job insecurity, self-esteem, and sociodemographic information. We used an analysis of variance or *t*-test to evaluate differences among the participants in terms of psychological distress, and Pearson’s r was calculated to examine correlations among psychological distress, self-esteem, and job insecurity among nurses in China. We also undertook hierarchical regression analysis to explore the mediating effects of self-esteem between job insecurity and psychological distress, according to Baron and Kenny’s method [38], and generated a standardized estimate (β), F, R², and R²-changes (ΔR²) for each step. Finally, we applied a nonparametric resampling method (2000 iterations) by running the PROCESS plugin in the SPSS Macro, as proposed by Preacher and Hayes, to examine the statistical significance of the mediating effect [39]. Before performing the regression analyses, we standardized all continuous variables to prevent multicollinearity. In all analyses, we considered any *p* value < 0.05 statistically significant.

## Results

### Sociodemographic information and distribution of psychological distress in categorical items

As Table 1 shows, the sample comprises 428 (92.6 %) women and 34 (7.4 %) men. The mean age of the participants was 31.19 ± 6.36 years, and their average years of work experience was 8.65 ± 7.12 months. Regarding duration of employment, 33.3 % had worked as nurses for 6–10 years, and 24.9 % for more than 10 years. In terms of education, 339 (73.4 %) of the nurses had earned a bachelor’s degree or higher; additionally, 71.9 % were married. Regarding job type, 40.5 % were permanent nurses. We observed no significant differences between psychological distress and sociodemographic information, save for years of work experience and job type.

**Table. 1:** Sociodemographic information and distribution of psychological distress in categorical items (*N* = 462)

Variable	n (%)	Psychological Distress (Mean ± SD)	t/F	P
Age			2.034	0.132
< 30 years	266(57.6)	24.03 ± 7.14		
31 ~ 40 years	150(32.5)	23.79 ± 7.01		
> 40 years	46(10.0)	21.72 ± 8.20		
Gender			3.835	0.051
Female	428(92.6)	23.57 ± 7.07		
male	34(7.4)	25.59 ± 8.87		
Years of working			4.217	0.006
< 3 years	108(23.4)	24.31 ± 7.19		
3 ~ 5 years	85(18.4)	23.08 ± 7.12		
6 ~ 10 years	154(33.3)	24.95 ± 7.09		
> 10 years	115(24.9)	22.00 ± 7.22		
Education level			1.590	0.208
Junior school or under	123(26.6)	23.73 ± 7.59		
Bachelor’s degree or above	339(73.4)	23.72 ± 7.10		
Economic conditions		*P* = 0.066	2.730	0.066
< 3000 ¥	102(22.1)	25.03 ± 7.97		
3000 ~ 5000 ¥	233(50.4)	23.65 ± 6.73		
> 5000 ¥	127(27.5)	22.80 ± 7.37		
Marital status			0.328	0.567
Single	117(25.3)	23.64 ± 7.29		
Married	345(74.7)	23.97 ± 7.05		
Job type			18.993	0.000
Permanent nurse	177(40.5)	24.96 ± 8.13		
Temporary nurse	285(59.5)	22.95 ± 6.94		

### Scores and correlations among self-esteem, job insecurity, and psychological distress

Table 2 shows descriptive results with respect to self-esteem, psychological distress, quantitative insecurity, and qualitative insecurity, the scores for which are 28.11 ± 3.24, 23.72 ± 7.22, 7.78 ± 2.50, and 6.81 ± 3.01, respectively. A total of 387 nurses reported high levels of psychological distress, with 83.8 % of sample scores being ≥ 16. Table 2 also presents the Pearson’s correlation results. Self-esteem negatively correlates with each of quantitative insecurity (*r* = − 0.278, *p* < 0.01), qualitative insecurity (*r* = − 0.251, *p* < 0.01), and psychological distress (*r* = − 0.374, *p* < 0.01). Psychological distress, on the other hand, positively correlates with quantitative insecurity (*r* = − 0.339, *p* < 0.01) and qualitative insecurity (*r* = − 0.282, *p* < 0.01).

**Table. 2:** Mean, Standard Deviations (SD) and Correlation of Variables

Variables	Mean ± SD	1	2	3	4
1 Self-esteem	28.11 ± 3.24	1			
2 Quantitative Insecurity	7.78 ± 2.50	-0.278**	1		
3 Quality Insecurity	6.81 ± 3.01	-0.251**	0.147**	1	
4 Psychological Distress	23.72 ± 7.22	-0.374**	0.339**	0.282**	1

** *P* < 0.01

### Results of hierarchical linear regression analysis

Our hierarchical linear regression analysis results (Table 3) show that the control variable (i.e., years of work experience) does not significantly correlate with psychological distress in step 1 (*R*^2^ = 0.001). Quantitative insecurity (β = 0.306, *p* < 0.001) and qualitative insecurity (β = 0.236, *p* < 0.001) are significantly positive predictors of psychological distress in step 2, explaining the 17.5 % variance in psychological distress. In the third step, self-esteem (β = −0.256, *p* < 0.001) does not predict psychological distress, and accounts for the 5.7 % variance in psychological distress. When we add self-esteem to the regression model, the relationship with each of quantitative insecurity (β = 0.306 to β = −0.242, *p* < 0.001), qualitative insecurity (β = 0.236 to β = 182, *p* < 0.001), and psychological distress significantly attenuates. Based on the fourth step of Baron and Kenny’s method, we can conclude that self-esteem partially mediates the relationship between the two dimensions of job insecurity and psychological distress.

**Table. 3:** Hierarchical Linear Regression Analysis Results

Variable	Psychological Distress		
	**Step3**	**Step2**	**Step3**
**Block 1**			
Years of Working	− 0.074	− 0.075	− 0.045
Job type	− 0.009	− 0.019	− 0.023
**Block 2**			
Quantitative Insecurity		0.306***	0.242***
Qualitative Insecurity		0.236***	0.182***
**Block 3**			
Self-esteem			− 0.256***
*** F***	1.269	24.386***	27.637***
***R*** ^***2***^	0.001	0.176	0.233
***△*** ***R*** ^***2***^	0.001	0.175	0.057

*** *P* < 0.001

Our bootstrapping results (Table 4) indicate that the path coefficient of the indirect effect of quantitative insecurity on psychological distress through self-esteem is 0.2432 (95 % confidence interval [CI]: 0.1437, 0.3826). The path coefficient of the direct effect of quantitative insecurity on psychological distress is 0.7368 (95 % CI: 0.4898, 0.9837); our bootstrapping results also show that the path coefficient of the indirect effect of quality insecurity through self-esteem is 0.1979 (95 % CI: 0.1085, 0.3103). The path coefficient of the direct effect of qualitative insecurity on psychological distress is 0.4821 (95 % CI: 0.2759, 0.6884). Thus, self-esteem is a partial mediator between each of quantitative insecurity and qualitative insecurity and psychological distress.

**Table. 4:** Self-esteem mediates the relationship between the dimensions of job insecurity and psychological distress

Independent Variable	Mediating Variable	Predictor	Pathway	Estimate	SE	95 % CI
						LL UL
Psychological distress	Self-esteem	Self-esteem: Quantitative Insecurity	a_1_	-0.3604	0.0581	-0.4746 -0.2462
		Psychological distress: self-esteem	b_1_	-0.6749	0.0968	-0.8652 -0.4846
		Indirect effect through self-esteem psychological distress: Quantitative Insecurity	a_1_b_1_	0.2432	0.0601	0.1437 0.3826
		Direct effect of Quantitative Insecurity on psychological distress	c_1_	0.7368	0.1257	0.4898 0.9837
		Self-esteem: Quality Insecurity	a_2_	-0.2706	0.0486	-0.3663 -0.1751
		Psychological distress: self-esteem	b_2_	-0.7201	0.0974	-0.9116 -0.5287
		Indirect effect through self-esteem psychological distress: Quality Insecurity	a_2_b_2_	0.1949	0.0515	0.1085 0.3103
		Direct effect of Quality Insecurity on psychological distress	c_2_	0.4821	0.1050	0.2759 0.6884

Note: a1 a2 means “the pathway of quantitative insecurity or quality insecurity could predict self-esteem, b1 b2 means “the pathway of self-esteem could predict psychological distress, c1 c2 means “the pathway of quantitative insecurity or quality insecurity could predict psychological distress

## Discussion

To the best of our knowledge, the current study is among the first to examine self-esteem as a potential mediator between job insecurity and psychological distress among Chinese nurses. Our main findings are as follows. The prevalence of psychological distress is 83.8 %, and the two dimensions of job insecurity positively correlate with psychological distress, whereas self-esteem negatively correlates with psychological distress. The results of mediation analysis show that self-esteem is a partial mediator between the two dimensions of job insecurity and psychological distress.

Additionally, the findings of the current study also enrich those of earlier surveys [14–16] and explain why not everyone that suffers from job insecurity experiences psychological distress. The prevalence of psychological distress reported in this study resembles that reported elsewhere [23]. We found the years of work experience highly relevant to psychological distress, in that the more years of work experience one has, the less psychological distress they are likely to have; this finding is also consistent with that of a previous survey [36]. In addition, we found that job type relates to psychological distress, in line with existing research results [36, 37]. We might also assert that temporary nurses are more likely to accept the inherent instability of contractual employment work than permanent nurses, and this acceptance might manifest as lower levels of psychological distress.

Our research revealed that among nurses, job insecurity and two dimensions of job insecurity positively correlate with psychological distress; this finding aligns with that of Kachi [40]. Other studies confirm that job insecurity has serious consequences not only on individual-level financial capacity, but also on psychosomatic health [15, 17, 41]. According to conservation of resource theory, when nurses feel that their job stability or continuity is threatened, they will invest considerable psychological, physiological, and emotional resources into maintaining and protecting their job, which is naturally vital to them. However, if their efforts go unrewarded, emotional exhaustion will occur. With the deepening of China’s healthcare reforms, more and more of China’s nurses will find themselves worried about their job stability, job rewards, and future career development, and this could lead to higher levels of psychological distress [3, 7, 9].

The current study found that self-esteem negatively correlates with psychological distress; this is a finding observed in previous studies [23, 27]. Feng et al. show that nurses with high positive self-image tend to have greater career adaptability resources, and consequently feel more enthusiastic about their job [23]. Nurses with low self-esteem, on the other hand, exhibit limited coping resources and perceive of their professional environment as uncontrolled; these conditions subsequently increase their risk for psychological distress. Simultaneously, nurses with low self-esteem often feel incompetent and worthless—in which case, they would strive to manage the negative effects of those beliefs, perhaps in dysfunctional ways. In this way, they can become even more distressed. Inversely, nurses who have a positive sense of self-esteem are more flexible, and they can accept their strengths and weaknesses. Considering the mitigating effects of self-esteem against psychological distress, it would appear that interventions that aim to bolster self-esteem—such as training or individual sessions involving a psychological counseling platform, setting up a “peer support team,” and creating a healthy organizational atmosphere—should be suggested.

This study is especially important, given that it is the first empirical attempt to reveal how self-esteem mediates between job insecurity and psychological distress among Chinese nurses. These results enrich previous findings about the explanatory mechanisms underlying the job insecurity–outcome relationship [22, 42]. The current findings indicate that modest levels of job insecurity can motivate employees to make constructive suggestions and change their current personal situation—steps that result in (and exemplify) successful coping. Conversely, high levels of job insecurity can erode an individual’s competence and their ability to manage a given situation, thereby resulting in adverse mental health effects [22]. Recent research suggests that context (e.g., a supportive, cooperative work climate) can influence an individual’s self-esteem, and that success or failure in various situations can result in fluctuations in an individual’s self-esteem [43, 44]. Meanwhile, the current findings also support the assertion that self-esteem, as a key intermediary process, affects employees’ reactions to job stressors. Considering the great importance of self-esteem against psychological distress—not to mention fluctuations in self-esteem—it appears that interventions by which to strengthen individual-level self-esteem would be of great benefit.

The current study, like all studies, has several limitations. First, due to its cross-sectional nature, we were unable to ascertain causal relationships among self-esteem, job insecurity, and psychological distress. Second, all data were collected using self-reported questionnaires, a process that incurs unavoidable reporting bias. Third, the participants were recruited from a tertiary hospital, and the sample size was small; these factors limit the generalizability of the results and weaken the statistical power. Future empirical longitudinal studies across hospitals of different classification will be required to establish causality and distinction among the variables.

## Conclusions

In summary, 83.8 % of the nurses participating in this study reported experiencing psychological distress. The two dimensions of job insecurity positively correlated with psychological distress, whereas self-esteem negatively correlated with psychological distress; mediation analysis showed that self-esteem was a partial mediator between the two dimensions of job insecurity and psychological distress. These findings call on health managers and researchers to take measures to strengthen self-esteem in the fight against psychological distress—measures that can take the form of various training and/or psychological well-being programs. 

## Data Availability

The datasets generated and analyzed for the current study are not publicly available due to IRB agreements but are available from the corresponding author on reasonable request.
